# T Cells Recognizing a Peptide Contaminant Undetectable by Mass Spectrometry

**DOI:** 10.1371/journal.pone.0028866

**Published:** 2011-12-14

**Authors:** Vedran Brezar, Slobodan Culina, Thomas Østerbye, François Guillonneau, Giovanni Chiappetta, Yann Verdier, Joelle Vinh, F. Susan Wong, Søren Buus, Roberto Mallone

**Affiliations:** 1 INSERM, U986, DeAR Lab Avenir, Saint Vincent de Paul Hospital, Paris, France; 2 Université Paris Descartes, Sorbonne Paris Cité, Faculté de Médecine, Paris, France; 3 Department of International Health, Immunology and Microbiology, Faculty of Health Sciences, University of Copenhagen, Copenhagen, Denmark; 4 Université Paris Descartes, Sorbonne Paris Cité, 3P5 Proteomics Facility, Paris, France; 5 Ecole Supérieure de Physique et de Chimie Industrielles de Paris, USR 3149 CNRS/ESPCI ParisTech, Paris, France; 6 Centre for Endocrine and Diabetes Sciences, Cardiff University, Cardiff, United Kingdom; 7 Assistance Publique – Hopitaux de Paris, Hôpital Cochin et Hôtel Dieu, Service de Diabétologie, Paris, France; Governmental Technical Research Centre of Finland, Finland

## Abstract

Synthetic peptides are widely used in immunological research as epitopes to stimulate their cognate T cells. These preparations are never completely pure, but trace contaminants are commonly revealed by mass spectrometry quality controls. In an effort to characterize novel major histocompatibility complex (MHC) Class I-restricted β-cell epitopes in non-obese diabetic (NOD) mice, we identified islet-infiltrating CD8+ T cells recognizing a contaminating peptide. The amount of this contaminant was so small to be undetectable by direct mass spectrometry. Only after concentration by liquid chromatography, we observed a mass peak corresponding to an immunodominant islet-specific glucose-6-phosphatase catalytic subunit-related protein (IGRP)_206-214_ epitope described in the literature. Generation of CD8+ T-cell clones recognizing IGRP_206-214_ using a novel method confirmed the identity of the contaminant, further underlining the immunodominance of IGRP_206-214_. If left undetected, minute impurities in synthetic peptide preparations may thus give spurious results.

## Introduction

Synthetic peptides produced by fluorenylmethyloxycarbonyl (FMOC) chemistry are widely used in a variety of research fields. In immunology, the main interest of peptides is that they can be synthesized to reproduce the amino acid sequence of antigenic epitopes, which are the protein fragments recognized by T cells upon processing and presentation by antigen-presenting cells (APCs). When tailored at an optimal amino acid length, they do not require uptake and processing by APCs, as they can directly bind major histocompatibility complex (MHC) Class I (8–11 amino acid length) and Class II (∼12–20 amino acid length) molecules on the APC surface for immediate presentation to CD8+ and CD4+ T cells, respectively.

Another major attractiveness is that production of peptides in suitable quantities and purity grades is much easier than that of recombinant antigens, which frequently carry over small protein or endotoxin contaminants from the bacterial, yeast or baculovirus systems where they are generated [1]. Thus, peptides are employed in different immunological studies both *in vitro*, to stimulate epitope-specific T cells, and *in vivo*, as immunizing agents in mice and as vaccination antigens in human trials [2].

Similarly, peptides are largely used in type 1 diabetes research to pinpoint the molecular targets of autoreactive T cells [3] which ignite recognition and destruction of insulin-producing pancreatic β cells [4]. Once identified, these epitopes are invaluable reagents to track the corresponding T cells and to neutralize their pathogenic potential through suitable tolerogenic delivery [5].

Despite these major advantages, even synthetic peptides cannot be produced to complete purity. This can frequently lead to misleading results, which are seldom reported in the literature [6], [7], [8]. To minimize these drawbacks, the workflow of peptide synthesis includes quality control steps, where purity is assessed through high performance liquid chromatography (HPLC) and mass spectrometry (MS) analyses before delivery to the final users. These analyses are frequently useful *a posteriori* to troubleshoot unexpected results, as contaminant peaks identified by MS can be scrutinized more carefully.

In this report, we describe an instance of a peptide contaminant which went undetected in several quality control MS analyses. Nonetheless, this minute contaminant was capable of eliciting peptide-specific T-cell responses *in vitro*. The troubleshooting procedure demonstrating the involvement of such contaminant in the observed results is described, including a novel technique to select antigen-specific CD8+ T-cell clones from infiltrated islets of non-obese diabetic (NOD) mice.

## Results

### Identification of a MHC Class I-restricted candidate epitope in NOD mice

**Figure 1:**
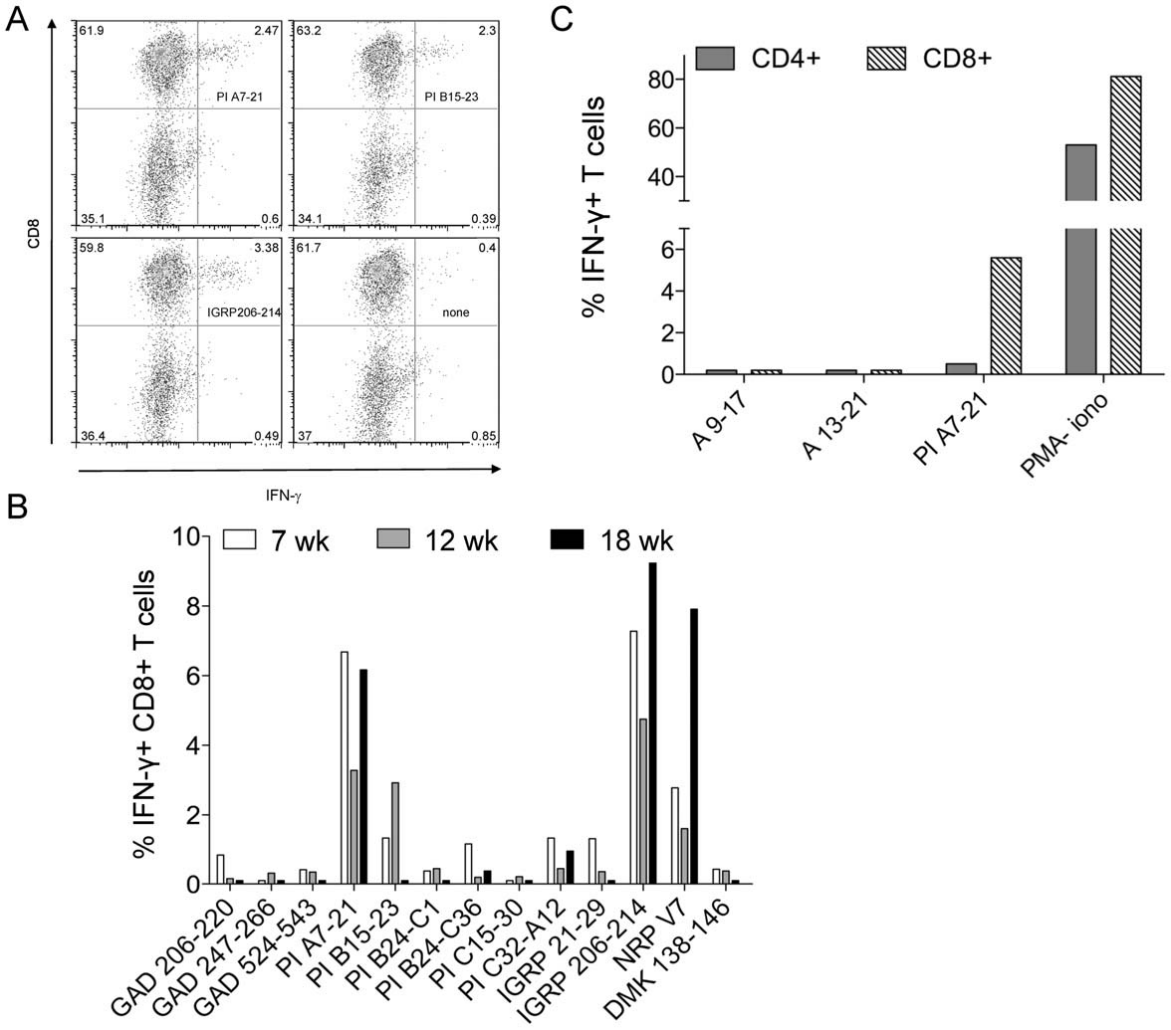
β-cell epitope-specific CD8+ T-cell IFN-γ responses in NOD islet infiltrates. (A) Representative staining showing IFN-γ responses of spontaneous NOD islet-infiltrating CD8+ T cells recalled *in vitro* with different peptides. Percent cells present in each quadrant are given. (B) IFN-γ-producing CD8+ T-cell responses in islets from NOD mice of different ages (n = 4/group) following *in vitro* peptide stimulation. (C) PI_A7-21_-specific responses in islet infiltrates originate from CD8+, but not from CD4+ T cells. The percent of IFN-γ+ CD4+ and CD8+ T cells after *in vitro* peptide recall (n = 4/group) is shown.

We set up to investigate the kinetics of CD8+ T-cell IFN-γ responses to a selected panel of previously described β-cell epitopes [9]. After isolating the islet infiltrates and culturing them for 7 d in the presence of IL-2 to enrich the population of CD8+ T cells, we observed that IFN-γ responses were very weak *vis à vis* most peptides, except for 3 of them: IGRP_206-214_, PI_B15-23_ and PI_A7-21_ (Fig. 1A–B). As reported [10], responses to IGRP_206-214_ and its mimotope NRP-V7 were detectable in NOD mice of different ages, while PI_B15-23_-reactive T cells were only detected until 12 weeks (Fig. 1B).

While IGRP_206-214_ and PI_B15-23_ are well described Class I K^d^-restricted epitopes [11], [12], PI_A7-21_ has been previously described as a Class II-restricted epitope [13]. To confirm that the observed PI_A7-21_-induced responses were Class I-restricted, we measured IFN-γ release upon peptide stimulation in both CD4+ and CD8+ T cells and found that only CD8+ T cells responded to PI_A7-21_ (Fig. 1C).

As the PI_A7-21_ peptide was unexpectedly long to bind to MHC Class I registries, we hypothesized that there could be a shorter variant either present in the peptide preparation or processed during peptide pulsing. However, testing of potential 9–11-mer core epitopes within the PI_A7-21_ sequence, either in spontaneous islet infiltrates or in splenocytes following peptide immunization, did not yield any similar IFN-γ response (data not shown). Indeed, we found two epitopes, PI_A9-17_ and PI_A13-21_, which showed significant T-cell responses after peptide immunization, but no spontaneous T-cell responses were observed in islet infiltrates (Fig. 1C).

### New peptide syntheses fail to reproduce the same reactivity

**Figure 2:**
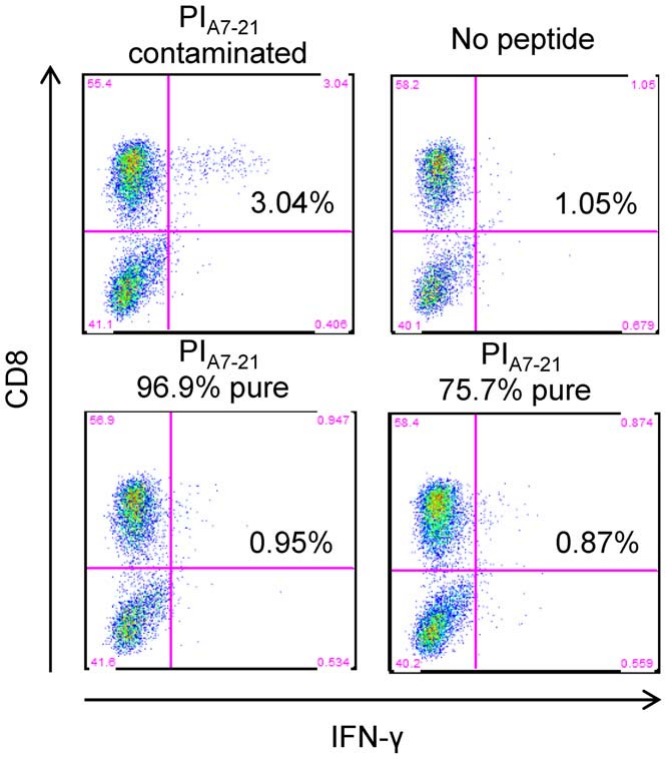
New PI_A7-21_ peptide syntheses fail to reproduce the same CD8+ T-cell responses. IFN-γ production by islet-infiltrating CD8+ T cells following *in vitro* stimulation with 3 different preparations of PI_A7-21_ peptide. The percent of IFN-γ+CD8+ cells is given.

The PI_A7-21_ peptide was resynthesized by another manufacturer at two different purity grades (75.7% and 96.9%). Neither of these new peptide preparations was capable of eliciting T-cell responses similar to our previous synthesis (Fig. 2). We thus hypothesized that the observed reactivity could be due to a contaminant present in the first peptide preparation.

### The original PI_A7-21_ preparation contains a PI_B15-23_ contaminant

**Figure 3:**
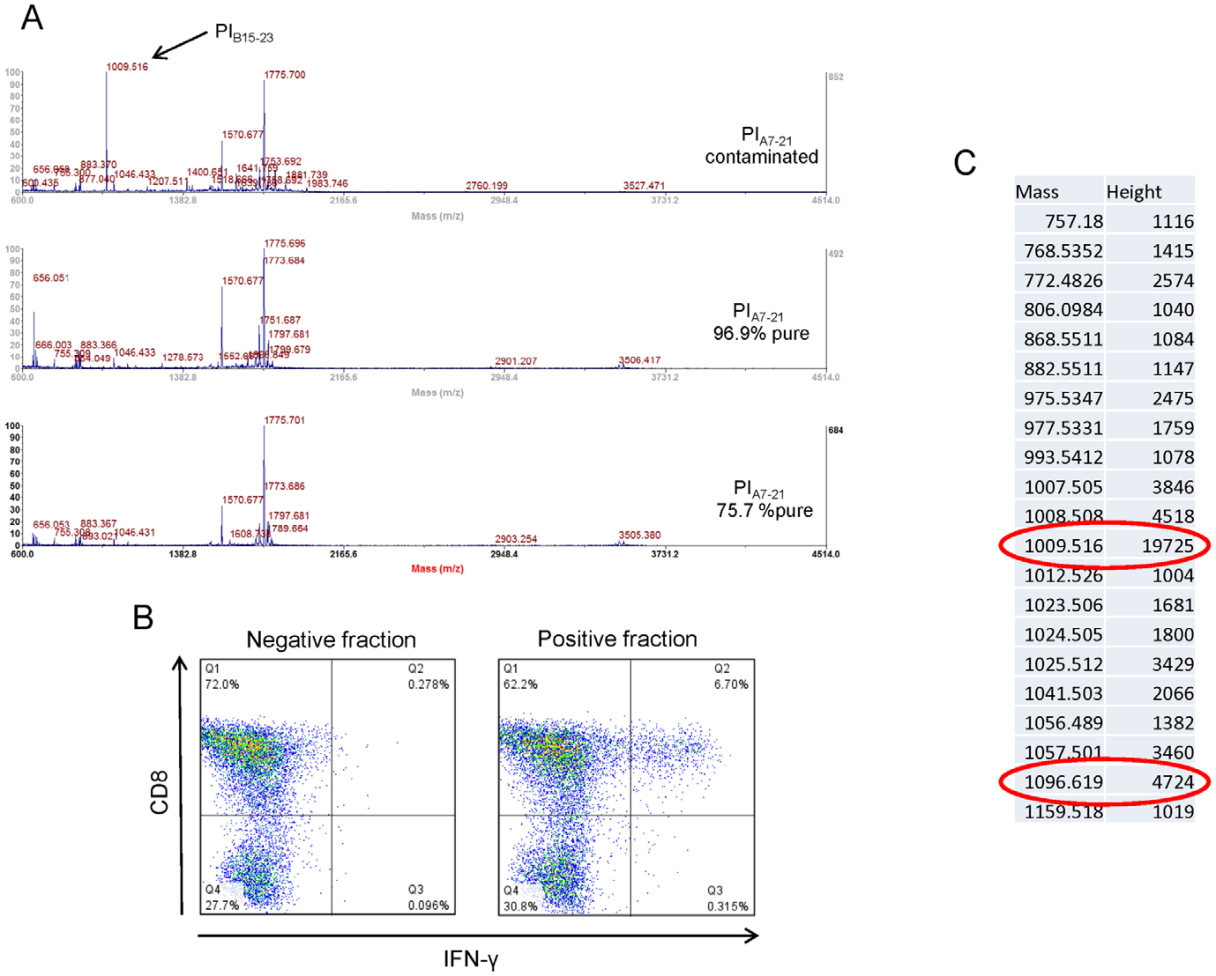
MS analysis of 3 different preparations of PI_A7-21_ peptide and nanochromatography fractionation of the contaminated PI_A7-21_ batch. (A) MS analysis: a contaminant with a mass peak of 1009,516 compatible with the MH+ adduct of PI_B15-23_ is detected in the contaminated PI_A7-21_ preparation. (B) One hundred microliters of a 50 µM solution of contaminated PI_A7-21_ peptide were subjected to nanochromatography and separated in 96 fractions. Each of these fractions was tested for its ability to induce IFN-γ responses in islet-infiltrating CD8+ T cells. Results obtained with one single positive fraction compared with one of the remaining 95 negative fractions are shown. (C) The T-cell-positive fraction identified was reanalyzed by MS. The obtained mass list shows two species at 1009,516 and 1096,619 m/z, compatible with the MH+ adduct of PI_B15-23_ and IGRP_206-214_, respectively.

A first MS analysis on the original PI_A7-21_ preparation highlighted a 1009,51 m/z mass peak compatible with the MH+ adduct of the PI_B15-23_ peptide (Fig. 3A). However, this contaminant could not explain the observed T-cell responses since, as shown in Fig. 1B, the PI_B15-23_ peptide does not induce T-cell responses as strong as contaminated PI_A7-21_ and is not recognized in islet infiltrates of older NOD mice. To definitely rule out the involvement of PI_B15-23_, we used the contaminated PI_A7-21_ preparation to stimulate PI_B15-23_-specific splenocytes from G9C8 transgenic mice expressing a PI_B15-23_-specific T-cell receptor [14]. No response was observed (data not shown), suggesting that the amount of PI_B15-23_ contaminant was insufficient to elicit T-cell responses. Thus, we hypothesized that there could be another peptide contaminating the original PI_A7-21_ preparation.

### Fractionation of the original PI_A7-21_ preparation reveals a mass peak corresponding to IGRP_206-214_ that elicits IGRP_206-214_-specific T-cell responses

**Figure 4:**
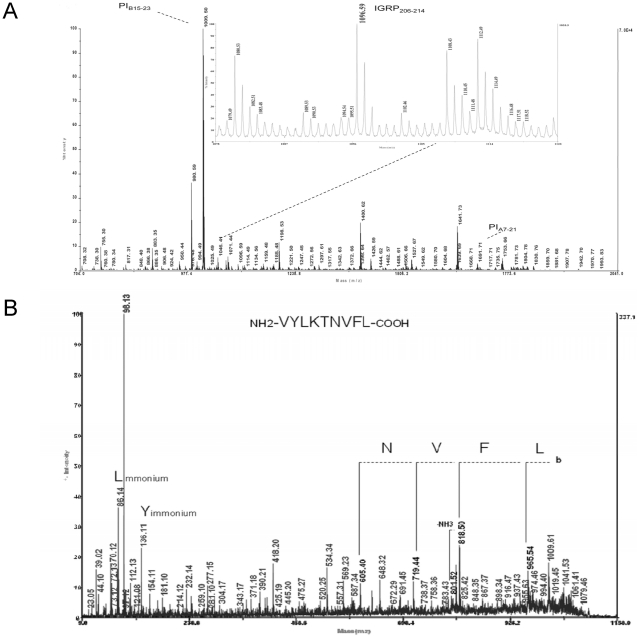
Partial sequencing of the IGRP_206-214_ contaminant by MS/MS. (A) MS analysis of the contaminated PI_A7-21_ preparation. (B) MS/MS analysis of the 1096.34 m/z compatible with the MH+ adduct of IGRP_206-214_.

To concentrate other possible peptide contaminant(s), we used HPLC fractionation. Ninety-six fractions were collected, concentrated 10-fold and subsequently tested for CD8+ T-cell IFN-γ responses from islet infiltrates. Only one of these 96 fractions was able to elicit a T-cell response (Fig. 3B). This fraction was reanalyzed by MS (Fig. 3C). The previously identified 1009.51 m/z peak corresponding to PI_B15-23_ was again detected, along with a 1096.62 m/z peak not previously visualized in the unfractionated PI_A7-21_ sample (compare with Fig. 3A). This peak is compatible with the MH+ adduct of the IGRP_206-214_ peptide. One further MS analysis attempted to conclusively identify IGRP_206-214_ but could only sequence part of the peptide (VYLKTNVFL) (Fig. 4). This is probably due to the extremely low amount of this contaminant.

**Figure 5:**
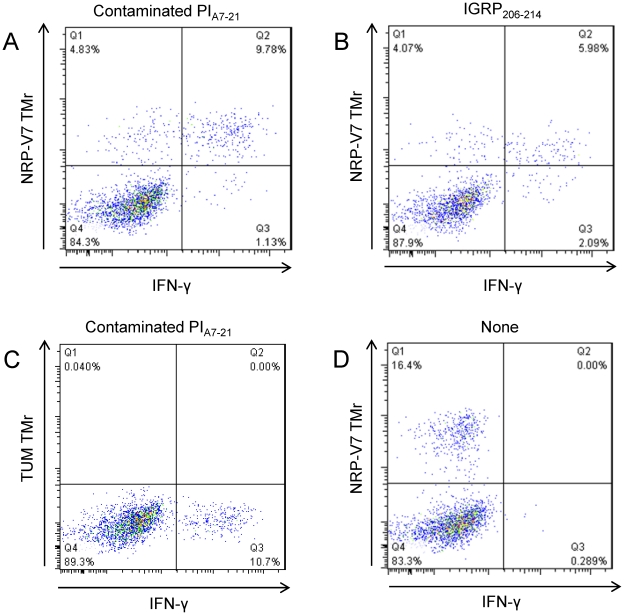
Islet-infiltrating CD8+ T-cells responding to contaminated PI_A7-21_ peptide are IGRP_206-214_-specific. Parallel IFN-γ and K^d^ TMr staining after stimulation with contaminated PI_A7-21_ or IGRP_206-214_ peptide. (A) Stimulation with contaminated PI_A7-21_ peptide and NRP-V7 K^d^ TMr staining. (B) Stimulation with IGRP_206-214_ peptide and NRP-V7 K^d^ TMr staining. (C) Stimulation with contaminated PI_A7-21_ peptide and TUM K^d^ TMr negative control staining. (D) Stimulation with peptide diluent alone and NRP-V7 K^d^ TMr staining.

To verify whether this minute IGRP_206-214_ peptide contaminant was responsible for the observed T-cell responses, we performed a parallel IFN-γ/TMr staining. We stimulated cultured islet infiltrates with the contaminated PI_A7-21_ preparation and double-stained them for intracellular IFN-γ and NRP-V7-loaded K^d^ TMrs (Fig. 5). The same CD8+ T cells which secreted IFN-γ in response to the contaminated PI_A7-21_ preparation also bound the NRP-V7 TMr (Fig. 5A), similar to IGRP_206-214_-stimulated cells (Fig. 5B). Staining with a control TUM-loaded K^d^ TMr or stimulation with peptide diluent alone did not yield any IFN-γ/TMr double-positive population (Fig. 5C–D). This experiment proves that the IGRP_206-214_ contaminant present in our PI_A7-21_ peptide sample is capable of eliciting IGRP_206-214_-specific CD8+ T-cell responses. We can also exclude the presence of other contaminants capable of stimulating CD8+ T cells, as ∼90% of contaminant-stimulated IFN-γ+ cells were also NRP-V7 TMr+ (Fig. 5A).

### CD8+ T-cell clones raised against the contaminated PI_A7-21_ peptide preparation are IGRP_206-214_- specific

**Figure 6:**
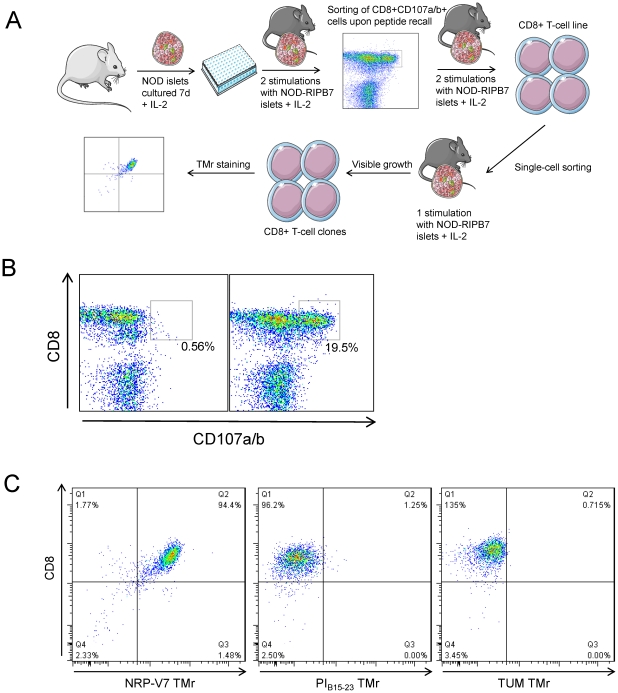
CD8+ T-cell clones raised against contaminated PI_A7-21_ peptide are IGRP_206-214_-specific. (A) Schematic of the T-cell expansion and cloning technique. (B) Sorting of epitope-reactive CD8+ T cells based on CD107a/b upregulation after *in vitro* peptide recall. Left plot shows negative control (no stimulation); right plot shows CD107a/b staining after stimulation with contaminated PI_A7-21_ peptide. (C) Representative TMr staining of one of the 3 T-cell clones generated. Staining for NRP-V7, PI_B15-23_ and negative control TUM K^d^ TMr is shown from left to right.

To finally prove that IGRP_206-214_ was the contaminant inducing T-cell responses, we expanded and cloned CD8+ T cells responding to our contaminated peptide. A diagram of the cloning procedure is shown in Fig. 6A. Total islet infiltrates were initially stimulated and expanded using islets from NOD-RIPB7 mice as a source of antigen. These islets further express the co-stimulatory molecule B7.1 [15]. The expanded infiltrates were subsequently challenged with the contaminated PI_A7-21_ preparation, stained with the cytotoxic granule marker CD107a/b [16] and sorted based on positive staining (Fig. 6B). Upon this peptide recall, 19.5% of islet-stimulated CD8+ T cells responded by CD107a/b upregulation. This fraction was single-cell-sorted. After one further round of stimulation with RIPB7 islets, 3 growing wells were identified out of the 60 seeded (5.0%). These 3 clones were tested for specificity by TMr staining (Fig. 6C). All of them stained uniformly positive for the NRP-V7 K^d^ TMr, but not for the PI_B15-23_ nor for the control TUM K^d^ TMr.

## Discussion

When we set out to investigate islet-infiltrating CD8+ T-cell responses to a panel of different β-cell peptides, we identified a candidate Class I-restricted epitope derived from the insulin A chain. New preparations of the same peptide did not however elicit CD8+ T-cell responses, and MS analyses on concentrated fractions revealed that the original preparation was contaminated with an IGRP_206-214_ epitope that was responsible for the observed responses. When validating epitope panels for T-cell recognition, it is therefore important that strong responses measured against a given peptide are reproduced on a separate preparation to rule out these pitfalls.

This IGRP_206-214_ contaminant was introduced during peptide synthesis and purification and not during laboratory manipulation. Indeed, peptide sampling from different portions of the vial including its very bottom gave similar T-cell responses, which would not be the case if cross-contamination from an IGRP_206-214_ preparation occurred during weighing of the lyophilized powder. Further supporting this conclusion, the original PI_A7-21_ preparation, although >90% pure, also contained traces of other peptides originated from insulin (i.e. PI_B15-23_) that were detected by MS. Impurities in synthetic peptides are mostly accounted for by products of deletion, additional residue incorporation, truncation and incomplete de-protection in solid phase synthesis [17]. This is not the case for our findings, since peptide contaminants corresponded to different amino acid sequences. Thus, the most likely possibility is that the peptide preparation was contaminated by improper handling of HPLC equipment during purification. Insufficient washing steps may have left traces of other peptides previously purified on the same column. Indeed, the manufacturer's log records revealed that PI_B15-23_ and IGRP_206-214_ preparations were purified on the same day, by the same operator using the same equipment.

The main interest of this case report is that the IGRP_206-214_ peptide contaminant remained undetected by conventional MS analyses, and a compatible mass peak could finally be revealed only by concentrating the sample 10-fold. This emphasizes the exquisite sensitivity of T cells in sensing even minute amounts of their cognate antigens. We used different technical approaches to identify this contaminant. To conclusively prove that IGRP_206-214_ was eliciting CD8+ T-cell responses, we produced clones selected based on upregulation of the cytotoxic granule marker CD107a/b [16] following peptide exposure. This approach is attractive for cloning epitope-specific CD8+ T cells for which the corresponding TMrs are not available. Indeed, very few CD8+ T-cell clones recognizing β-cell epitopes have been isolated from spontaneous islet infiltrates in the NOD mouse [18], [19], [20].

Our results further emphasize the impressive immunodominance of the IGRP_206-214_ epitope within the NOD islet infiltrates. They also exemplify how a fraction of IGRP_206-214_-specific CD8+ T cells infiltrating the islets is of very high avidity, as it was capable of responding to minute amounts of peptide.

In conclusion, high purity peptides may hide traces of contaminating peptides potentially leading to experimental pitfalls. Functional T-cell assays are extremely sensitive to these impurities, which may be missed by MS analysis. While this sensitivity makes T-cell assays particularly vulnerable, it can also provide means to conclusively identify these contaminants.

## Materials and Methods

### Peptides

Proinsulin (PI)_B24-C1_ (FFYTPMSRRE), PI_C15-30_ (SFGDLQTLALEVARQK), islet-specific glucose-6-phosphatase catalytic subunit-related protein (IGRP)_21-29_ (TYYGFLNFM), glutamic acid decarboxylase (GAD)_206-220_ (TYEIAPVFVLLEYVT), GAD_247-266_ (NMYAMLIARYKMFPEVKEKG), and GAD_524-543_ (SRLSKVAPVIKARMMEYGTT) were produced by Schafer-N. PI_B24-C36_ (FFYTPMSRREVED), PI_C32-A12_ (KRGIVDQCCTSICS) and non-contaminated PI_A7-21_ peptides (CTSICSLYQLENYCN) were produced by GL Biochem. All these peptides were >80% pure. IGRP_206-214_ (VYLKTNVFL), PI_B15-23_ (LYLVCGERG), dystrophia myotonica kinase (DMK)_138-146_ (FQDENYLYL) and contaminated PI_A7-21_ (CTSICSLYQLENYCN) were produced by “manufacturer X” at >90% purity.

### Mice

NOD mice were bred and housed in specific pathogen-free conditions. NOD mice were crossed with C57BL/6J-RIP-B7-1 mice [15] to obtain a first generation (F1) of offspring that was used to prepare islet feeders [18]. G9C8 transgenic mice expressing the T-cell receptor from a G9C8 CD8+ clone recognizing the PI_B15-23_ peptide [14] were also used. All experiments were conducted according to ethic guidelines. This study was approved by the local ethics committee for animal experimentation (*Comité régional d'éthique pour l'expérimentation animale*, approval number: P2.AL.116.09).

### Preparation of pancreatic islet cell infiltrates

Mice were sacrificed and pancreatic islets isolated after perfusion with 0.75 mg/ml collagenase P (Roche), as described [21], [22]. To measure antigen-specific CD8+ T-cell responses, islets were isolated and cultured for 7 d in the presence of 50 U/ml recombinant human interleukin (IL)-2 (R&D). Infiltrating cells were then collected and analyzed.

### Flow cytometry

Cells were stained with PE-labeled K^d^ tetramers (TMrs) synthesized by the National Institute of Health Tetramer Core Facility and loaded with NRP-V7 (a mimotope of the IGRP_206-214_ epitope; KYNKANVFL) [11] and control TUM peptide (KYQAVTTTL). Peptide-stimulated interferon (IFN)-γ responses were measured using intracellular IFN-γ staining after incubation for 5 h in the presence of 10 µM peptide and 10 µg/ml Brefeldin A (Sigma). Phorbol myristate acetate (PMA)/ionomycin (1 µg/ml/each) were used as a polyclonal positive control stimulus. CD8-Alexa Fluor 700 (clone 53-6.7; eBioscience), CD4-PerCP (clone RM4-5; BD), IFN-γ-PE (clone XMG1.2; BD), CD45-FITC (clone 30-F11; BD) and CD107a/b-Alexa Fluor 647 (clone 1D4B; eBioscience) were used. For intracellular IFN-γ staining, Cytofix/Cytoperm kit (BD) was used.

### Generation of epitope-specific CD8+ T-cell clones

Isolated islets from prediabetic NOD mice were cultured for 7 d in the presence of 50 U/ml recombinant human IL-2.

#### Restimulation

On the 7^th^ day of culture, 8–10 irradiated (2,500 rad) islets/well from NOD-RIPB7 (F1) mice were added and cultured for 7 d in the presence of 50 U/ml IL-2. Fresh IL-2 (50 U/ml final concentration) was added every 3 d. Fresh medium was added when needed (every 7 d throughout the cloning). Fourteen days after the addition of NOD-RIPB7 islets, another restimulation was performed in the same way.

#### Sorting of epitope-specific CD8+ T cells

14 d after the second restimulation, recovered cells were stained with CD107a/b in the presence of 10 µM peptide and 0.7 µM monensin (Sigma) for 5 h at 37°C. One hundred CD8+CD107a/b+ cells/well were sorted in 96-well V-bottom plates containing 10 fresh irradiated islets from NOD-RIPB7 mice as feeders. IL-2 (50 U/ml) was added and replenished every 3 d at the same final concentration. Fresh medium was added approximately every 7 d.

#### Restimulation

The first round of restimulation was performed as before. For the second round, cells were transferred to 96-well flat-bottom plates and the number of feeder islets increased to 20/well.

#### Single-cell sorting

14 d after the second restimulation, one viable [7-aminoactinomycin D (7AAD)-negative] cell was sorted in each well of a 96-well V-bottom plate, containing 10 NOD-RIPB7 islets/well. IL-2 was added as before and restimulation repeated once as before until noticeable growth.

### Nanochromatography

Peptide solutions (5 µl, 10 mM) were diluted 1∶200 in 10% acetonitrile (ACN; Carlo Erba), 0.1% trifluoroacetic acid (TFA; Pierce). These preparations were either directly analysed by matrix-assisted laser desorption/ionisation (MALDI) MS as described below or separated and concentrated by reverse phase nanochromatography as follows. Ten microliters of diluted peptides were injected and separated with an Ultimate 3000 series HPLC (Dionex). Injected peptides were trapped using solvent A (0.1% TFA, 2% ACN) at 30 µl/min loading flow for 3 min in a C18 trap column (Acclaim reverse phase C18 pepmap 100 phase, 5 µm particles, 100 Å pores, 5 mm length, 300 µm internal diameter). The microvalve then oriented the elution to back flow (300 nl/min) the peptides towards the analytical column (C18 pepmap 100, 3 µm particle, 100 Å pores, 15 cm length, 75 µm i.d.) with a gradient rising from 10% solvent B (80% ACN, 20% solvent A) at microvalve switch to 60% B in 34 min. Fractions (20 s/each, i.e. 100 nl) were deposited in 96 wells of a 384-well plate using a Probot fraction collector (Dionex). In order to increase the concentration of collected peptides in each well, the injection/fractionation was repeated 20 times and collected in the same 96 plate positions to accumulate identical fractions. Fractions were subsequently dehydrated in a speed vacuum concentrator (Eppendorf). After solubilization in 5 µl 10% ACN 0.1% TFA, each well represented a 10-fold concentration factor of the initial peptide solution. Half microliter of each fraction was deposited on a MALDI target to perform MS analysis.

The initial peptide dilution or the concentrated fractions were mixed 1∶1 on a MALDI target plate with 1 µl of 5 mg/ml of α-cyano-4-hydroxycinnamic acid (CHCA) matrix (Laser Biolabs) in 70% ACN 0.1% TFA and 60 nM Glu-fibrinopeptide (Sigma) as internal standard and analysed using a 4800 MALDI time-of-flight (TOF) TOF analyser (ABSciex).

### MS

Spectra acquisition and processing were performed using the 4000 series explorer software (ABSciex) version 3.5.28193 build 1011 in positive reflectron mode at fixed laser fluency with low mass gate and delayed extraction. External plate calibration was performed using 4 calibration spots at each corner of the plate. Additional internal calibration was performed using Glu-fibrinopeptide (m/z 1570.677). For each acquisition, 10 increments of 50 spectra in the range of m/z 600 to 4500 were acquired at a 200 Hz laser shot frequency. Five-hundred MS spectra per sample were summed and processed to obtain monoisotopic values from isotope clusters with a raw spectra s/n ratio of 20.

### MS/MS

In each MS spectrum, the 12 most abundant peaks were selected for fragmentation, starting with the least abundant. A cut-off was applied at a minimum s/n of 20. Neighboring precursors within 200 resolution were excluded. 1000 MS/MS spectra per precursor were summed by increments of 50. Processing included baseline subtraction and Stavitsky Golay smoothing with 3 points across peak and a polynomial order of 4. Generated peaklists reflected monoisotopic values from isotope clusters with a minimum s/n ratio of 22.

### Peptide identification

Peak lists were submitted to an in-house mascot (Matrix science) version 2.2 search engine [23]. The Swiss-Prot database release 2010_05 (516603 sequences; 181919312 residues) was used with restriction to mammalia (60,000 sequences). No enzymes were selected as cleavage specificity. Parent and fragment mass tolerances were respectively set to 50 ppm and 0.3 Da; variable modification (oxidation) of methionines was allowed. A filter was applied to the search in order to reduce false positives and matching redundancies of the same peptide in several hits. All matches above 5% risks of random matching were eliminated (p<0.05). Peptide lists only included proteins matching the same set of peptide and, if shared subsets were present, at least one non-shared peptide was required. The minimal peptide score was 25. With these parameters, the minimal protein score was 50.
